# Estimating the proportion of people with chronic hepatitis B virus infection eligible for hepatitis B antiviral treatment worldwide: a systematic review and meta-analysis

**DOI:** 10.1016/S2468-1253(20)30307-1

**Published:** 2020-11-14

**Authors:** Mingjuan Tan, Ajeet S Bhadoria, Fuqiang Cui, Alex Tan, Judith Van Holten, Philippa Easterbrook, Nathan Ford, Qin Han, Ying Lu, Marc Bulterys, Yvan Hutin

**Affiliations:** a Department of HIV/AIDS and Global Hepatitis Programme, WHO, Geneva, Switzerland; b All India Institute of Medical Sciences, Rishikesh, Uttarakhand, India; c Department of Medicine, National University Health System, Singapore; d Singapore

## Abstract

**Background:**

In 2016, of the estimated 257 million people living with chronic hepatitis B virus (HBV) infection worldwide, only a small proportion was diagnosed and treated. The insufficiency of information on the proportion of people infected with HBV who are eligible for treatment limits the interpretation of global treatment coverage. We aimed to estimate the proportion of people with chronic HBV infection who were eligible for antiviral treatment worldwide, based on the WHO 2015 guidelines.

**Methods:**

In this systematic review and meta-analysis, we searched Medline, EMBASE, and the Cochrane databases from Jan 1, 2007, to Jan 31, 2018, for studies describing HBsAg-positive people in the population or health-care facilities. We extracted information from published studies using a standardised form to estimate the frequency of cirrhosis, abnormal alanine aminotransferase (ALT), HBV DNA exceeding 2000 IU/mL or 20 000 IU/mL, presence of HBeAg, and eligibility for treatment as per WHO and other guidelines as reported in the studies. We pooled proportions through meta-analysis with random effects. The study was registered with PROSPERO, CRD42020132345.

**Findings:**

Of the 13 497 studies, 162 were eligible and included in our analysis. These studies included 145 789 participants. The pooled estimate of the proportion of cirrhosis was 9% (95% CI 8–10), ranging from 6% (4–8) in community settings to 10% (9–11) in clinic settings. Examining the proportion of participants who had characteristics used to determine eligibility in the WHO guidelines, 1750 (10·1%) of 17 394 had HBV DNA exceeding 20 000 IU/mL, and 20 425 (30·8%) of 66 235 had ALT above the upper limit of normal. 32 studies reported eligibility for treatment according to WHO or any other guidelines, with a pooled estimate of eligibility at 19% (95% CI 18–20), ranging from 12% (6–18) for studies in community settings to 25% (19–30) in clinic settings.

**Interpretation:**

Many studies described people with HBV infection, but few reported information in a way that allowed assessment of eligibility for treatment. Although about one in ten of the 257 million people with HBV infection (26 million) might be in urgent need of treatment because of cirrhosis, a larger proportion (12–25%) is eligible for treatment in accordance with different guidelines. Future studies describing people with HBV infection should report on treatment eligibility, according to broadly agreed definitions.

**Funding:**

WHO and US Centers for Disease Control and Prevention.

## Introduction

WHO estimates that, in 2015, 257 million people worldwide were living with hepatitis B virus (HBV) infection (defined as being positive for HBsAg).^1^ 68% of these people were living in the WHO-defined African and Western Pacific regions.^1^ Chronic HBV infection, if left untreated, can progress to cirrhosis and hepatocellular carcinoma.^2^ WHO also estimates that in 2015, 887 000 people died of chronic HBV infection worldwide.^1^ Antiviral agents suppress HBV replication, prevent progression to cirrhosis, and reduce the risk of hepatocellular carcinoma and liver-related deaths.^3^ In 2015, WHO produced its first guidelines on prevention, care, and management of chronic HBV infection, with a focus on low-income and middle-income countries.^4^ Key recommendations included the use of non-invasive tests for the staging of liver disease; prioritisation of treatment for people at greatest risk of disease progression and mortality, including those with cirrhosis, or, among people without cirrhosis, patients with the combination of high HBV DNA levels (>20 000 IU/mL) and persistently raised alanine aminotransferase (ALT). These guidelines recommended the preferred use of nucleos(t)ide analogues with a high genetic barrier to resistance (ie, tenofovir or entecavir). Other professional guidelines from the Asian-Pacific Association for the Study of the Liver (APASL),^5^ the American Association for the study of Liver Diseases (AASLD),^6^ and the European Association for the Study of the Liver (EASL)^7^ made comparable recommendations for treatment eligibility, although with some differences in thresholds of ALT and HBV DNA for treatment (appendix p 2). The 2012 EASL guidelines^7^ were revised in 2017,^8^ expanding the criteria for eligibility. Although many treatment guidelines are available, and the cost of treatment has decreased by 85% between 2004 and 2016, access to and uptake of testing and treatment remains scarce, especially in many low-income and middle-income countries. WHO estimated that in 2016, 27 million (or 10·5%) of people estimated to be HBsAg positive were diagnosed, and, of people diagnosed, 4·5 million (17%) were receiving antiviral treatment.^9^

**Research in context**

**Evidence before this study**

WHO estimated that, in 2016, among the 257 million people living with chronic hepatitis B virus (HBV) infection worldwide, only 10·5% had been diagnosed and of those, only 17% were receiving treatment. We searched PubMed for systematic reviews published between Jan 1, 2007 and Jan 31, 2018, using “hepatitis B” and “treatment”, but did not identify any estimates, based on empirical data, on the proportion of people with chronic HBV infection eligible for treatment worldwide. The only studies available were studies reporting people with HBV infection, recruited either in the community or in health-care facilities. Although a few of these studies reported the proportion of HBV-infected people who were eligible for treatment, many did not. In the absence of an estimate of the denominator of HBV-infected people eligible for treatment, as per current treatment guidelines, treatment coverage estimates are hard to interpret.

**Added value of this study**

We systematically reviewed studies describing people with HBV infection to provide a description of their clinical status and assess eligibility. We found that there are many missed opportunities to estimate the proportion of people eligible for treatment. Most studies did not report data that could be used to examine eligibility in a consistent format. We also provided a first estimate of the proportion of people eligible for HBV treatment. About 10% of people with HBV infection are eligible for urgent treatment because of cirrhosis. In addition, overall, between 12% and 25% of people might be eligible for treatment because of either cirrhosis or the combination of raised ALT and viral replication.

**Implications of all the available evidence**

More opportunities should be seized to estimate the proportion of people who are eligible for HBV treatment. Simple descriptive studies should include estimates of the proportion of participants who are eligible for treatment. Additional efforts are needed to generate disaggregated estimates of the proportion of people eligible for HBV treatment by type of recruitment (community or health-care setting), age, sex, ethnicity, and geographical areas, so as to better understand who is eligible. This information could be used to optimise testing approaches, so that population groups with the highest yield of eligible patients could be tested as a priority. Patients with HBV-related cirrhosis (around 10% people with HBV infection) should be prioritised for treatment initiation. These patients can be treated in the absence of HBV DNA testing.

According to the eligibility criteria of the various treatment guidelines, only a subset of people with chronic infection are in need of treatment, and this subset varies by population, region, and setting. However, no estimates are available for the proportion of people who are infected with HBV who meet these treatment eligibility criteria in different regions.^1^ Hence, the extent to which the number of people initiated on treatment corresponds to the need is not known. As a consequence, estimates of the treatment gap are difficult to interpret. If available, estimates of the proportion of people positive for HBsAg who were eligible for treatment would allow for better analyses of antiviral treatment gaps and targets, which would support planning of treatment programmes.

To address this knowledge gap, we systematically reviewed the literature to estimate the proportion of HBsAg-positive people who met eligibility criteria for treatment. Our overall goal was to better inform future data collection on eligibility criteria to inform progress in treatment scale-up at national and regional levels. Our aim was to estimate the proportion of people with chronic HBV infection who were eligible for antiviral treatment worldwide, based on the WHO 2015 guidelines.^4^ We aimed to estimate the proportion of treatment-eligible people according to other professional society guidelines (appendix p 2) and describe the availability of data that would allow assessment of eligibility for hepatitis B antiviral treatment (ie, ALT, HBV DNA, cirrhosis, or liver fibrosis status) in published studies.

## Methods

### Search strategy and selection criteria

In this systematic review and meta-analysis, we searched MEDLINE, Embase, and the Cochrane database for studies published from Jan 1, 2007, to Jan 31, 2018, that described population of HBsAg-positive people in terms of criteria that can be used to assess eligibility for treatment (ie, ALT, HBV DNA, or cirrhosis; appendix p 3). We also screened bibliographies of relevant articles. We included articles in English, Chinese, French, Spanish, Portuguese, Russian, Korean, and Japanese; although in the final analysis, after inclusion and exclusion criteria were applied, only studies in English, Chinese, French, and Spanish met the inclusion criteria. Two reviewers (MT and ASB) screened titles and abstracts, with inclusions verified by a third reviewer (YH). Disagreements were resolved by discussion and consensus (appendix p 4).

### Data analysis

We included studies that reported data on a population of HBsAg-positive people and at least one of the following: liver fibrosis or cirrhosis (F0 to F4, irrespective of test type), data on abnormal ALT levels (more than upper limit of normal of the laboratory, as defined by the study), and data on HBV DNA. We excluded studies focusing entirely or mostly on children (defined as people <18 years); studies with fewer than 20 participants; studies of the complications of HBV infection; case series of liver imaging or biopsy without information on selection of participants; studies focusing on people with HIV, hepatitis C virus, hepatitis D virus co-infection (co-infections affect eligibility and are a minority of all HBV infections^10^, ^11^) or focusing on people with other characteristics that would have led to the study population being representative of a subset of the population of people with HBV infection (eg, people who were immunosuppressed);^1^ studies focusing mainly on participants with a primary condition other than HBV infection; and studies restricted to participants already known to be eligible for therapy and studies that included people who were on treatment. If multiple publications reported the same study population, we included the more recent analysis. For meta-analyses and reviews that included results from several primary studies, we obtained individual studies and extracted data from the primary source if not already included.

We assessed method quality through possible sources of selection and information bias. For selection bias, we reviewed the study design (eg, cohort, cross-sectional, or case control), data collection procedures (eg, prospective or retrospective), and the type of recruitment (eg, from statistical samples in the community, to non-statistical samples in the community, to recruitment in health-care facilities). For information bias, we extracted information on methods used to assess liver fibrosis and viral replication. For methods to assess fibrosis, we considered the range between non-invasive methods (including clinical assessment) to liver biopsy. When more than one method was used to assess liver fibrosis, we extracted data on the best method used, as determined by the assessor. For viral replication, we extracted information about whether HBV DNA or HBeAg was used. We then stratified results for all these determinants of study quality.

We extracted data on summary estimates from the selected articles and verified the information with a second reviewer (appendix p 5). We estimated the proportion of people with individual characteristics by which their treatment eligibility would be determined using WHO criteria (ie, cirrhosis or no cirrhosis, replication based on HBV DNA >20 000 IU/mL, or HBeAg, and presence of abnormal ALT according to the laboratory). We calculated means, medians, and proportions, and corresponding 95% CIs for estimates. We used the total number of studies reporting the information as the denominator to calculate CIs. We considered guidelines of the WHO, APASL, EASL, and AASLD to evaluate eligibility. We extracted information as to whether the study reported a proportion of people eligible for treatment as per any guidelines and reported this proportion.

For two specific outcomes (cirrhosis and eligibility per any guidelines), we did a DerSimonian-Laird random effects meta-analysis with Freeman-Tukey double arcsine transformation. We generated forest plots to estimate pooled estimates, *I*^2^ statistics to describe heterogeneity among studies, funnel plots to explore publication bias, and bubble plots to explore variations according to specific characteristics. We stratified the analysis according to year of publication, design of the study from which we extracted cross-sectional data on people with HBV infection (cohort, case-control, and cross-sectional), data collection procedure (prospective, retrospective, and cross-sectional), settings in which study participants had been recruited (outpatient, inpatient, population-based, community, and special groups), and WHO region.

All analyses were done in Epi-Info (version 7.2) and Stata (version 13). The meta-analysis was done using the metaprop, metabias, metafunnel, and metareg programmes in Stata. The protocol of the meta-analysis was registered in PROSPERO (CRD42020132345).

### Role of the funding source

The funder who supported WHO for this study had no role in study design, data collection, data analysis, data interpretation, or writing of the report. The first, second, and corresponding author had full access to all of the data and the final responsibility to submit for publication.

## Results

**Figure 1:**
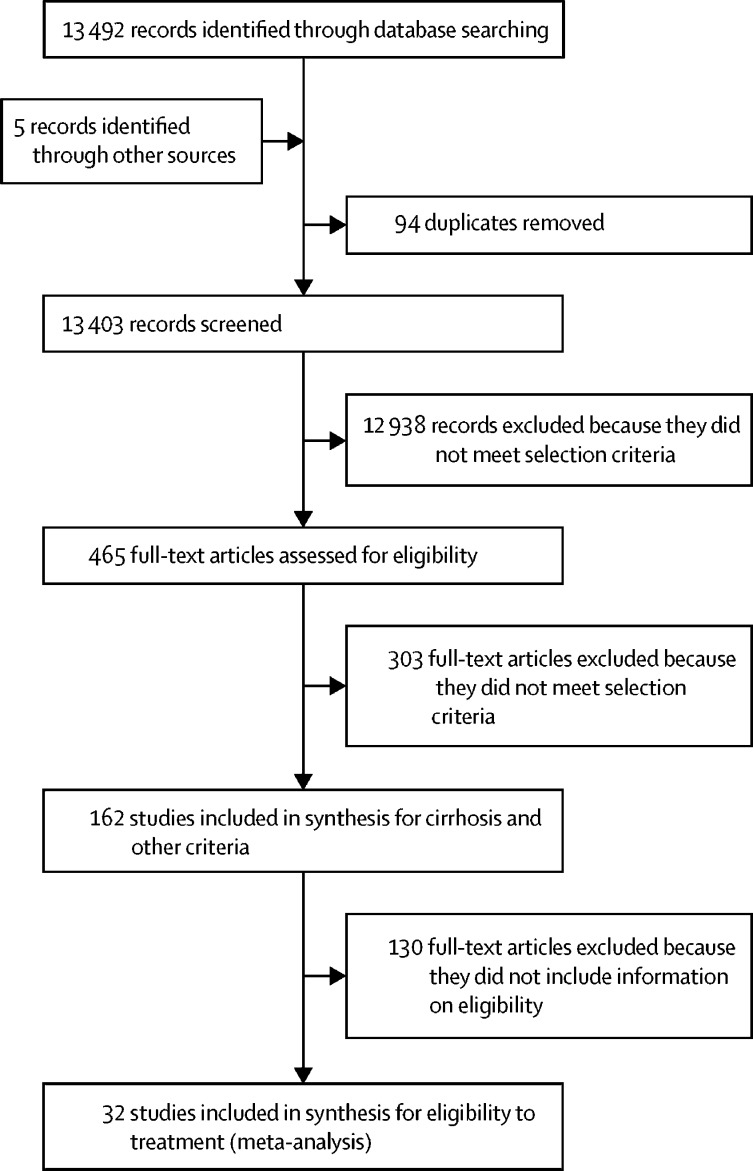
Study selection

**Figure 2:**
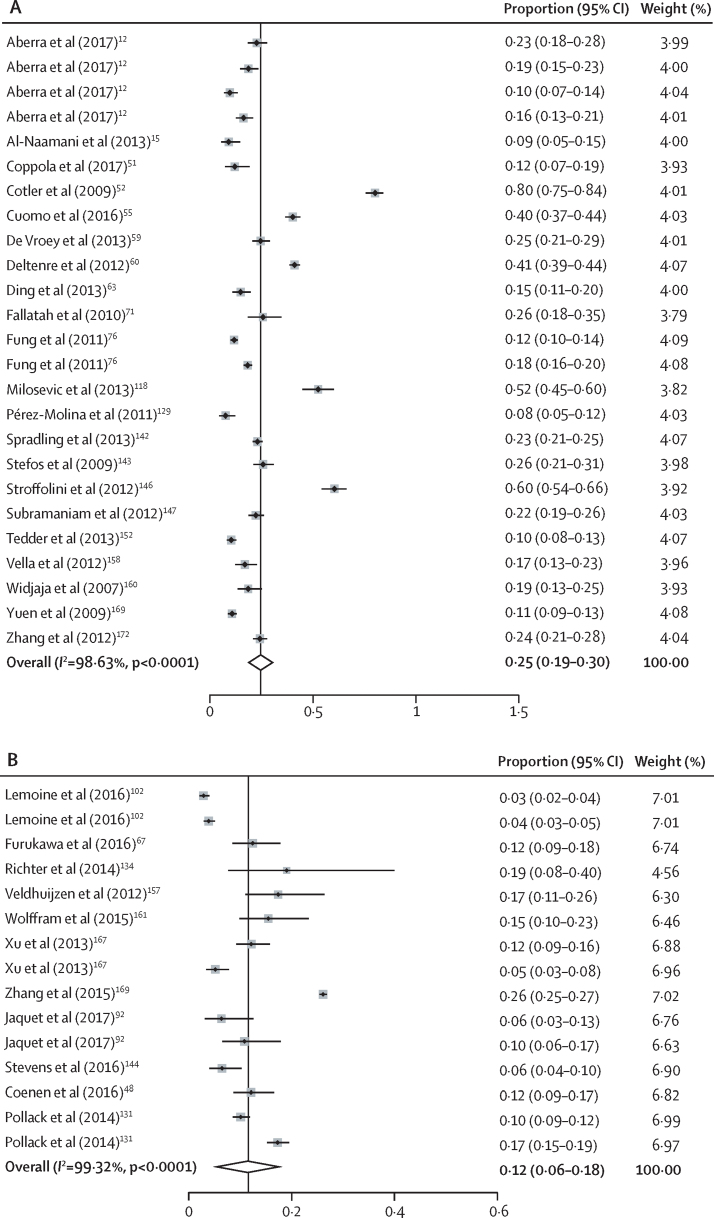
Pooled estimates of the proportion of HBsAg-positive people eligible for treatment as per any of the known guidelines in studies done in health-care facilities (A) or in the community (B) Cotler SJ and colleagues^52^ is an outlier. However, its removal in a sensitivity analysis did not affect the estimate substantially. Studies that appear more than once reported eligibility criteria using more than one set of guidelines.

The database search returned 13 492 records, and another five records were identified through other sources. 13 403 records were unique, after removal of duplicates, and of these, 465 were selected for full-text screening. Review of the full-text articles led to the exclusion of 303, and the inclusion of 162 articles in the analysis (Figure 1, Figure 2).^12^, ^13^, ^14^, ^15^, ^16^, ^17^, ^18^, ^19^, ^20^, ^21^, ^22^, ^23^, ^24^, ^25^, ^26^, ^27^, ^28^, ^29^, ^30^, ^31^, ^32^, ^33^, ^34^, ^35^, ^36^, ^37^, ^38^, ^39^, ^40^, ^41^, ^42^, ^43^, ^44^, ^45^, ^46^, ^47^, ^48^, ^49^, ^50^, ^51^, ^52^, ^53^, ^54^, ^55^, ^56^, ^57^, ^58^, ^59^, ^60^, ^61^, ^62^, ^63^, ^64^, ^65^, ^66^, ^67^, ^68^, ^69^, ^70^, ^71^, ^72^, ^73^, ^74^, ^75^, ^76^, ^77^, ^78^, ^79^, ^80^, ^81^, ^82^, ^83^, ^84^, ^85^, ^86^, ^87^, ^88^, ^89^, ^90^, ^91^, ^92^, ^93^, ^94^, ^95^, ^96^, ^97^, ^98^, ^99^, ^100^, ^101^, ^102^, ^103^, ^104^, ^105^, ^106^, ^107^, ^108^, ^109^, ^110^, ^111^, ^112^, ^113^, ^114^, ^115^, ^116^, ^117^, ^118^, ^119^, ^120^, ^121^, ^122^, ^123^, ^124^, ^125^, ^126^, ^127^, ^128^, ^129^, ^130^, ^131^, ^132^, ^133^, ^134^, ^135^, ^136^, ^137^, ^138^, ^139^, ^140^, ^141^, ^142^, ^143^, ^144^, ^145^, ^146^, ^147^, ^148^, ^149^, ^150^, ^151^, ^152^, ^153^, ^154^, ^155^, ^156^, ^157^, ^158^, ^159^, ^160^, ^161^, ^162^, ^163^, ^164^, ^165^, ^166^, ^167^, ^168^, ^169^, ^170^, ^171^, ^172^, ^173^

Of the 162 studies included (table 1), 50 (31%) were published in 2007–11, and 112 (69%) were published in 2012–18. From these 162 studies, all 145 789 participants were included from all WHO regions (median number of HBsAg-positive participants per study 285, IQR 123–818), with the largest median number from the Western Pacific (476 [IQR 226–1394] and smallest from the Americas (185 [144–610]). Participants had a mean age of 41 years (SD 8·8), and cohorts were comprised of a mean of 41·9% (22·5) women.

**Table 1:** Characteristics of HBsAg-positive people

	**Studies available (N=162)**	**Number of HBsAg-positive participants in individual studies (N=162)**				**Age in individual studies (n=89)**			**Proportion of females in individual studies (n=154)**	
		Mean (SD)	Median (IQR)	Range	Total	Mean (SD)	Median (IQR)	Range	Mean (SD)	Range
**Year of publication**										
2007–11	50 (31%)	728 (1075)	285 (114–1106)	25–4405	36 408	40·6 (7·4)	40·2 (35·7–46·3)	26–55	34·6 (19·3)	0–100
2012–18	112 (69%)	1094 (2473)	261 (134–841)	21–13 979	109 381	41·3 (9·3)	41·0 (34·5–48·4)	23–69	43·6 (23·0)	0–100
**Recruitment setting**										
Outpatient clinic	54 (31%)	720 (1719)	295 (143–576)	50–12 016	31 568	40·3 (6·8)	40·0 (35·3–45·0)	29–56	35·2 (17·6)	0–100
Inpatients	68 (39%)	757 (1719)	282 (139–691)	25–13 210	47 709	41·0 (9·1)	40·7 (34·0–48·0)	26–69	43·1 (22·7)	13–100
Population-based*	11 (6%)	1433 (2423)	400 (168–1542)	34–8875	15 760	44·9 (3·2)	45·0 (41·3–48·4)	40–49	54·1 (18·0)	34–100
Community†	21 (12%)	2146 (3487)	382 (139–2682)	21–13 979	42 928	49·3 (7·5)	50·2 (45·0–55·2)	37–64	48·5 (14·1)	34–100
Special groups‡	20 (12%)	455 (924)	156 (90– 811)	64–2903	9106	33·5 (7·8)	29·2 (28·4–39·8)	26–52	54·0 (34·7)	0–100
**WHO region**										
Americas	34 (12%)	899 (2085)	185 (144–610)	40–12 016	27 872	41·4 (5·0)	40·7 (38·0–45·4)	31–49	42·3 (21·7)	13–100
African	46 (17%)	852 (1840)	387 (146–756)	75–12 016	34 114	37·5 (8·0)	36·4 (31·0–41·9)	25–56	44·0 (27·5)	0–100
Eastern Mediterranean	33 (12%)	359 (709)	220 (114–473)	21–2078	11 125	37·8 (6·0)	36·9 (34·0–41·1)	23–50	34·6 (15·7)	13–100
European	51 (19%)	775 (1878)	291 (135–675)	21–13 210	35 806	42·6 (7·7)	42·0 (38·0–48·0)	26–57	40·6 (20·8)	0–100
Southeast Asia	40 (15%)	610 (704)	399 (170–691)	64–3760	19 434	40·8 (9·0)	41·0 (31·9–47·0)	26–56	39·0 (21·9)	0–100
Western Pacific	72 (26%)	1425 (2580)	476 (226–1394)	21–13 979	98 360	43·7 (9·6)	44·0 (38·2–49·1)	26–68	46·0 (22·1)	0–100
**Design**§										
Cohort	51 (31%)	1062 (1857)	441 (167–1200)	59–12 016	48 875	42·4 (9·5)	41·0 (36·8–45·5)	27–69	44·3 (21·5)	0–100
Case control	3 (2%)	523 (497)	289 (NA)	65–1215	1569	33·8 (NA)	32·8 (NA)	33–33	27·0 (7·3)	17–32
Cross-sectional	107 (67%)	964 (2295)	239 (111–602)	21–13 979	95 160	40·7 (8·3)	40·4 (34·8–48·9)	23–57	42·8 (23·2)	0–100
**Data collection**¶										
Prospective	88 (55%)	849 (1397)	300 (112–1139)	21–8875	66 585	41·7 (8·9)	41·0 (35·5–48·9)	25–64	43·0 (24·1)	0–100
Retrospective	44 (27%)	1244 (2725)	356 (180–655)	93–13 210	49 772	40·5 (7·1)	40·0 (35·5–45·9)	26–56	42·6 (19·7)	5–100
Cross-sectional	29 (18%)	1012 (2861)	170 (94–406)	34–13 979	29 346	40·4 (10·8)	40·4 (32·8–48·0)	23–69	38·3 (21·8)	0–100
Overall	162 (100%)	972 (2141)	285 (123–818)	21–13 979	145 789	41·0 (8·8)	41·0 (34·6–47·0)	23–69	41·9 (22·5)	0–100

Data are n (%), mean (SD), or median (IQR), unless stated otherwise. HBV=hepatitis B virus. NA=not applicable.

* Studies of individuals not seeking care sampled using statistically representative methods.

† Studies of individuals not seeking care, but recruited without statistically representative methods.

‡ For example, injection drug users and men who have sex with men.

§ Design of the study from which was extracted information on a population of people with HBV infection. Total exceeds the number of studies because some studies fit more than one category.

¶ One study with missing information on data collection procedure.

11 (6%) studies were based on representative samples of the general population; the remainder were based on either convenience samples in the population (ie, community; n=21 [12%]), outpatient clinics (n=54 [31%]), inpatients (n=68 [39%]) or specific population groups (eg, prisoners or migrants; n=20 [12%]). Only ten studies (6%) included a substantial proportion of pregnant women, representing 2897 (2·0%) of the total population of 145 789 HBsAg-positive people.

Most studies (n=107 [67%]) were cross-sectional in design. 51 (31%) were cohort studies and three (2%) were case-control studies. Data collection was mostly prospective (n=88 [55%]), with the remainder being retrospective (n=44 [27%]) or done in context of a cross-sectional survey (n=29 [18%]).

Methods used to assess liver fibrosis or presence of cirrhosis included clinical assessment (n=43 [26%]), other non-invasive methods (ie, clinical, ultrasound, or transient elastography n=102 [63%]), ultrasound (n=59 [36%]), transient elastography (n=20 [12%]), and biopsy (n=61 [37%]; table 2). 56 (35%) studies used more than one method to assess liver fibrosis.

**Table 2:** Liver fibrosis status and assessment methods used in studies describing HBsAg-positive people

	**Proportion of HBsAg-positive participants with selected stages of liver fibrosis**			**Proportion of studies using different methods used to assess liver fibrosis**				
	F2 (n=16)*	F3 (n=16)*	F4 (cirrhosis; n=104)*	Clinical assessment (43 studies)	NIM (102 studies)	Ultrasound (59 studies)	Transient elastography (20 studies)	Biopsy (61 studies)
**Year of publication**								
2007–11 (n=50)	26% (24–27)	20% (18–21)	11·0% (10·6–11·4)	29% (16–41)	67% (54–79)	43% (30–57)	4% (1–13)	41% (27–54)
2012–18 (n=112)	18% (16–19)	16% (15–17)	8·5% (8·2–8·7)	25% (18–34)	61% (52–70)	33% (25–42)	15% (10–23)	36% (27–45)
**Recruitment setting**								
Outpatient clinic (n=54)	31% (28–34)	33% (30–35)	9·5% (9·2–9·9)	46% (32–59)	62% (55–80)	45% (33–58)	13% (6–21)	51% (38–65)
Inpatients (n=68)	30% (29–32)	24% (22–25)	13·9% (13·5–14·4)	26% (17–38)	57% (46–69)	32% (22–44)	13% (7–23)	43% (32–54)
Population-based (n=11)†	1% (<1–2)	3% (2–4)	22·0% (20·0–24·0)	9% (2–38)	50% (19–81)	18% (5–46)	9% (2–38)	9% (2–38)
Community (n=21)‡	..	9% (5–13)	9·6% (8·9–10·2)	14% (5–35)	85% (69–100)	52% (32–72)	10% (3–29)	24% (11–45)
Special groups (n=20)	6% (2–9)	8% (2–14)	7·7% (6·9–8·6)	50% (24–76)	60% (38–81)	40% (21–61)	20% (8–42)	25% (11–47)
**WHO region**								
Americas n=34)	5% (2–8)	6% (2–9)	7·3% (6·9–7·6)	41% (26–58)	74% (59–88)	44% (29–61)	6% (2–19)	68% (51–81)
African (n=46)	12% (10–13)	15% (13–17)	8·1% (7·8–8·4)	30% (17–43)	57% (43–72)	30% (18–44)	21% (12–35)	45% (31–60)
Eastern Mediterranean (n=33)	15% (11–19)	4% (2–6)	12·8% (12·1–13·4)	30% (17–47)	63% (47–80)	39% (25–56)	12% (5–27)	51% (35–67)
European (n=51)	37% (33–42)	18% (14–21)	14·2% (13·7–14·9)	35% (22–48)	54% (40–67)	33% (21–46)	10% (4–21)	54% (40–67)
Southeast Asia (n=40)	5% (2–8)	6% (2–9)	15·3% (14·7–15·8)	37% (22–52)	63% (49–78)	41% (27–57)	5% (2–16)	60% (44–75)
Western Pacific (n=72)	26% (24–28)	21% (19–23)	8·6% (8·2–8·8)	23% (13–33)	59% (43–71)	37% (27–48)	7% (3–15)	40% (28–51)
Overall	21% (19–23)	18% (17–19)	9·5% (9·3–9·7)	26% (19–33)	63% (55–70)	36% (29–44)	12% (8–17)	37% (30–45)

Data are % (95% CI). NIM=non-invasive methods, include clinical criteria, ultrasound, or transient elastography.

* Number of studies available that estimated the proportion of people with this specific stage of liver fibrosis. Only a minority of studies reported data on the proportion of persons with F2 and F3 fibrosis.

† Studies of individuals not seeking care sampled using statistically representative methods.

‡ Studies of individuals not seeking care but recruited without statistically representative methods.

Overall, 6563 (9·5%) of 69 129 participants had F4 liver fibrosis (cirrhosis). The meta-analysis (appendix p 12) estimated that 9% (95% CI 8–10) of participants had cirrhosis, which was not influenced by the assessment method (appendix p 13). There was a higher proportion of participants with higher prevalence of cirrhosis in larger studies (appendix p 14). In regression with random effect, the proportion of participants with cirrhosis ranged from 6% (95% CI 4–8) in community settings to 10% (9–11) in clinic settings (appendix p 17). We were unable to extract an estimate of the proportion of patients with decompensated cirrhosis.

HBeAg or HBV DNA, or both, were used to assess viral replication (table 3). The proportion of studies with information on HBeAg was higher (n=122 [75%] of 162 studies) than for HBV DNA (n=41 [25%] for 2000 IU/mL threshold and 20 [12%] for the 20 000 IU/mL threshold). Overall, 19 459 (17·7%) of 109 577 participants were HBeAg positive, which was highest in the Western Pacific (19·6% [95% CI 19·1–19·9]) and lowest in the Americas (12·8% [95% CI 12·3–13·1]). 10 101 (28·2%) of 35 826 participants had HBV DNA of more than 2000 IU/mL, which was highest in Europe (31·4% [95% CI 29·6–33·2]) and lowest in the Americas (12·9% [12·4–13·5]). 1750 (10·1%) of 17 394 participants had HBV DNA of more than 20 000 IU/mL, which was highest in southeast Asia (29·2% [24·7–33·7]) and lowest in the Americas (6·4% [6·0–6·9]). The proportions of people with HBeAg and high HBV DNA were also higher in studies done from 2007–11 than 2012–18. Of 162 studies, 80 (49%) reported data on ALT. 20 425 (30·8%) of 66 235 participants had ALT higher than the upper limit of normal, with this number being highest in Europe (54·9% [95% CI 54·0–55·9]) and lowest in the Western Pacific (30·7% [30·4–31·1]). Because information about liver fibrosis, replication, and abnormal ALT were reported in aggregate numbers and without cross tabulation, it was not possible to infer eligibility from these individual criteria or how eligibility was assessed in combination.

**Table 3:** HBV replication and abnormal ALT status in HBsAg-positive people

	**Proportion of people with HBeAg (n=122)**	**HBV DNA**		Abnormal ALT*	
		Proportion of people with >2000 IU/mL (n=41)	Proportion of people with >20 000 IU/mL (n=20)	Proportion of people with ALT>ULN (n=80)	Proportion of people with ALT >2 times ULN (n=18)
**Year of publication**					
2007–11 (n=50)	20·4% (19·9–21·3)	43·2% (41·8–44·5)	30·0% (17·3–42·7)	35·6% (34·9–36·2)	26·0% (24·5–27·5)
2012–18 (n=112)	16·2% (15·9–16·5)	25·8% (25·3–26·3)	10·0% (9·5–10·4)	28·9% (28·5–29·3)	8·3% (7·9–8·7)
**Recruitment setting**					
Outpatient clinic (n=54)	18·7% (18·2–20·2)	18·7% (18·1–19·4)	9·3% (8·8–9·8)	36·9% (36·3–37·5)	10·1% (9·7–10·6)
Inpatients (n=68)	21·4% (20·9–21·7)	35·2% (33·8–36·5)	22·7% (20·9–24·5)	45·4% (44·6–46·2)	10·3% (9·4–11·2)
Population-based (n=11)†	19·3% (18·5–20·0)	6·5% (5·1–7·9)	..	28·9% (28·2–29·6)	6·9% (3·7–10·0)
Community (n=21)‡	13·5% (13·0–13·9)	41·1% (40·2–41·9)	21·0% (18·1–23·9)	29·2% (28·4–30·1)	7·6% (5·3–9·9)
Special populations (n=20)	16·8% (15·9–17·8)	14·5% (13·4–15·7)	13·7% (12·0–15·4)	16·0% (15·1–16·9)	8·3% (4·9–11·7)
**WHO region**					
Americas (n=34)	12·8% (12·3–13·1)	12·9% (12·4–13·5)	6·4% (6·0–6·9)	34·7% (34·0–35,5)	6·4% (6·0–6·9)
African (n=46)	12·2% (11·8–12·6)	14·9% (14·3–15·4)	8·2% (7·7–8·6)	32·7% (32·0–33·3)	7·1% (6·7–7·5)
Eastern Mediterranean (n=33)	15·1% (14·3–15·8)	22·1% (19·4–24·8)	21·3% (18·1–24·4)	33·6% (32·3–35·0)	7·6% (5·8–9·4)
European (n=51)	13·7% (13·3–14·0)	31·4% (29·6–33·2)	13·3% (11·5–15·1)	54·9% (54·0–55·9)	13·3% (12·3–14·4)
Southeast Asia (n=40)	18·2% (17·7–18·6)	14·5% (12·7–16·2)	29·2% (24·7–33·7)	53·6% (52·5–54·7)	15·6% (14·5–16·8)
Western Pacific (n=72)	19·6% (19·1–19·9)	24·8% (24·3–25·4)	6·6% (6·2–7·0)	30·7% (30·4–31·1)	10·7% (10·2–11·2)
Overall (N=162)	17·7% (17·5–17·9)	28·2% (27·7–28·7)	10·1% (9·6–10·5)	30·8% (30·5–31·2)	11·0% (10·6–11·4)

Data are % (95% CI). HBV=hepatitis B virus. ALT=alanine aminotransferase. ULN=upper limit of normal.

* ALT levels were defined as abnormal using the criteria of the individual studies and their laboratories.

† Studies of individuals not seeking care sampled using statistically representative methods.

‡ Studies of individuals not seeking care, but recruited without statistically representative methods.

The proportion of participants eligible for treatment was reported using various criteria and only in a small number of studies (table 4). According to EASL 2012 criteria (eight studies), 830 (19·3%) of 4300 participants were eligible for treatment. This number was highest in the Americas (36% [95% CI 33–39]) and in Europe (32% [29–35]) and lowest in the African region (18% [16–19]). According to AASLD criteria (four studies), 246 (8%) of 3030 participants were eligible. This number was highest in the Americas (19% [95% CI 13–24]), with no data from the Eastern Mediterranean or European regions. According to EASL 2017 criteria (two studies, both in Africa), 75 (18%) of 410 participants were eligible. According to WHO criteria (two studies, both in Africa), 40 (10%) of 410 participants were eligible. Overall, of 162 studies, only 32 (20%) assessed eligibility for treatment using WHO or any other guidelines. The pooled estimate of eligibility according to any criteria was 19% (95% CI 18–20), but there was considerable heterogeneity. Stratified analysis led to pooled estimates of treatment eligibility of 12% (6–18) among studies done in community settings and 25% (19–30) in studies from health facilities (hospital or clinic settings). Studies done in health-care facilities that included older participants tended to have a lower proportion of eligibility (p=0·006; appendix p 15). The funnel plots did not suggest any significant publication bias with respect to proportion of eligibility, whether in health-care facilities or the community (appendix p 16).

**Table 4:** Eligibility of HBsAg-positive people for treatment according to guidelines

	**AASLD (n=4)**		**EASL 2012 (n=8)**		**EASL 2017 (n=2)**		**WHO (n=2)**		Other*(n=16)	
	Studies available	Eligible patients	Studies available	Eligible patients	Studies available	Eligible patients	Studies available	Eligible patients	Studies available	Eligible patients
**Year of publication**										
2007–11 (n=50)	2 (4%)	12% (10–14)	1 (2%)	18% (16–20)	..	..	..	..	4 (8%)	15% (13–17)
2012–18 (n=112)	2 (2%)	3% (2–4)	7 (6%)	20% (18–21)	2 (2%)	18% (15–22)	2 (2%)	10% (7–13)	12 (11%)	20% (19–21)
**Recruitment setting**										
Outpatient (n=54)	2 (4%)	12% (11–14)	4 (7%)	19% (17–20)	1 (2%)	23% (18–27)	1 (2%)	10% (6–13)	7 (13%)	19% (17–20)
Inpatients (n=68)	..	..	2 (3%)	29% (25–33)	1 (1%)	23% (18–27)	1 (1%)	10% (6–13)	9 (13%)	21% (20–22)
Population based (n=11)†	2 (18%)	3% (2–4)	1 (9%)	4% (3–5)	..	..	..	..	..	..
Community (n=21)‡	1 (5%)	6% (3–9)	1 (5%)	19% (2–26)	..	..	..	..	3 (14%)	13% (10–16)
Special groups (n=20)	..	..	1 (5%)	12% (6–18)	1 (5%)	6% (2–11)	1 (5%)	10% (4–16)	1 (5%)	12% (8–16)
**WHO region**										
American (n=34)	1 (3%)	19% (13–24)	2 (6%)	36% (33–39)	..	..	..	..	4 (12%)	22% (20–24)
African (n=46)	1 (2%)	3% (2–4)	5 (11%)	18% (16–19)	2 (4%)	18% (14–22)	2 (4%)	10% (7–13)	4 (9%)	15% (13–17)
Eastern Mediterranean (n=33)	..	..	1 (3%)	19% (2–26)	..	..	..	..	3 (9%)	16% (14–18)
European (n=51)	..	..	4 (8%)	32% (29–35)	..	..	..	..	6 (12%)	22% (20–24)
Southeast Asia (n=40)	1 (2%)	6% (3–10)	2 (5%)	22% (18–25)	..	..	..	..	5 (12%)	18% (16–19)
Western Pacific (n=72)	2 (3%)	11% (9–12)	3 (4%)	25% (24–27)	..	..	..	..	8 (11%)	17% (16–18)
Overall (N=162)	4 (2%)	8% (7–9)	8 (5%)	19% (19–20)	2 (1%)	18% (14–22)	2 (1%)	10% (7–13)	16 (10%)	19% (18–20)

Data are n (%) or % (95% CI). AASLD=American Association for the study of Liver Diseases. EASL=European Association for the Study of the Liver.

* Studies used guidelines other than AASLD, EASL, or WHO, or used mixed guidelines to document treatment eligibility.

† Studies of individuals not seeking care sampled using statistically representative methods.

‡ Studies of individuals not seeking care but recruited without statistically representative methods.

## Discussion

Among the 162 studies identified that described people with chronic HBV infection, few provided information that could estimate the proportion of people eligible for treatment. However, among the HBsAg-positive participants, approximately 9% had cirrhosis, 10% had HBV DNA exceeding 20 000 IU/mL, and roughly a third had raised ALT levels on at least one occasion. Estimates of treatment eligibility according to WHO or other guidelines varied between 12% in the community and 25% in clinical settings.

Most studies included some assessment of the stage of liver fibrosis, and according to the data generated through various methods, approximately one in ten people would have a liver fibrosis stage of F4, corresponding to cirrhosis. The methods used in the studies differed, were sometimes based on clinical criteria, and sometimes included tests that can be operator dependent or non-standardised (eg, ultrasound). Some studies relied on ultrasound to diagnose cirrhosis.^2^ Surface nodularity can be a reliable marker of advanced liver disease in patients with chronic liver disease undergoing liver biopsy, with sensitivity and specificity of more than 90%.^174^ In a case series without portal hypertension, the positive predictive value of ultrasound to diagnose cirrhosis was 68%.^175^ However, in the absence of features of portal hypertension, such as varices, ascites, or splenomegaly, the sensitivity can be low.^176^, ^177^, ^178^ WHO guidelines recommend non-invasive tests of acceptable diagnostic accuracy, such as the Fibrosis-4, AST to platelet ratio index, or transient elastography (as well as clinical assessment).^4^ Our meta-regression analysis did not suggest that this estimate was substantially influenced by the assessment methods. Assessment techniques not considered gold standard might be harder to use to differentiate between earlier stage of liver fibrosis, whereas cirrhosis might lead to changes that might be easier for these various tests to capture. In clinical practice and for individual decision making, the reliability of ultrasound and other less validated tests to diagnose cirrhosis is unclear. However, on a population level, the bubble plot analysis suggests that the assessment methods did not influence the proportion of people with cirrhosis. The combination of these techniques provides some order of magnitude of the proportion of HBV-infected individuals who might require treatment on the basis of cirrhosis, suggesting that roughly one in ten patients with HBV infection would be in that category. There are differences in the frequency of cirrhosis by region, for which we are unable to disentangle selection bias, information bias, and actual differences caused by other factors. The higher frequency of cirrhosis in the European region, for example, might be a result of an information bias. In Europe, there could be a more systematic use of reliable methods of staging and ascertainment of cirrhosis (eg, liver biopsies or transient elastography); however, there could be also more patients with other chronic liver diseases (eg, alcohol or metabolic syndrome).

Although the majority of studies reported on the proportion of people positive for HBeAg, fewer than half used HBV DNA as an assessment of viral replication, and only 20% reported data using the 20 000 IU/mL threshold used by WHO guidelines. Cost might explain, to some extent, why this technique is not more widely available; however, cost is not the only obstacle. Information on ALT was also not available in about half of the studies. Most importantly, although information might be available on liver fibrosis, replication (HBeAg or HBV DNA), and abnormal ALT as separate factors, studies reported data on an aggregated basis and without cross-tabulation. This aggregated data prevented a posteriori calculation of the proportion of people eligible for treatment during this meta-analysis.

Only a minority of studies estimated the proportion of patients eligible for treatment. Given the diversity of guidelines, these studies also reported a diversity of estimates. Our meta-analysis estimate suggested that overall, 19% (95% CI 18–20) of people infected with HBV would need treatment, whereas the pooled estimates of treatment eligibility ranged from 12% (6–18) in the community to 25% (18–20) in clinical settings. However, the representativeness of these studies and the diversity of the criteria used suggest these estimates should be interpreted with caution. The frequency of eligibility was not higher in the highly endemic regions of Africa or the Western Pacific. Differences in the results might be secondary to information bias. Higher frequency of eligibility in the higher income countries of the Americas might be because of diagnosis criteria and more frequent testing services, such as liver biopsies, that result in not only evaluating the degree of liver fibrosis, but also the degree of inflammation (AASLD recommends treatment of people with moderate inflammation or F2 liver fibrosis or greater). In our review, the largest use of biopsies was reported from the Americas. We observed that studies done in health-care facilities that recruited older participants tended to have a lower proportion of eligibility for treatment; this could be because many older participants might already be receiving treatment and a larger proportion of older people in the inactive phase of HBV infection.

This systematic review has several limitations. First, we attempted to retrieve information on eligibility from studies that were not designed to generate it. The information required involved tests that are not often used in low-income and middle-income countries (eg, reliable liver fibrosis assessment techniques or HBV DNA) and data were not always presented in a way that allowed for calculation of eligibility. In addition, because these studies were not done for this objective, information was not presented in a format that reflected the standard algorithm for decision making. As a result, we often missed the proportion that met criteria for treatment initiation, including the presence of cirrhosis, and of those without cirrhosis, the proportion with raised HBV DNA levels combined with ALT levels. Second, the diversity of treatment guidelines made it more difficult to estimate a single proportion of patients eligible for treatment because this varies according to the guidelines. Also, our study cannot be used to compare the proportion of people eligible for treatment according to different guidelines. Such analyses should be done in studies containing individual patient records. Third, we meta-analysed aggregated data from studies that did not disaggregate their study participants by subgroup, such as ethnic group, age, sex, or various other causes of liver diseases. Therefore, the variation in the estimate that we observed according to age, for example, is at the study level and could be explained by selection, information bias, and other factors. As an example of this limitation, we were unable to assess whether other chronic liver diseases (eg, alcohol or metabolic syndrome) would explain the frequency of cirrhosis by regions. Fourth, antiviral treatment for HBV infection is generally lifelong, and so a decision to treat (except for those with identified cirrhosis) is often made on the basis of several visits and repeat investigations over a period of time (eg, persistently raised aminotransferase levels). This is reflected in WHO, EASL,^7^, ^8^ and AASLD^6^ guidelines. Because most studies were cross-sectional in design, only a single timepoint was available to assess eligibility. Finally, we did not estimate the proportion of women eligible for prophylaxis during pregnancy, because criteria differ^179^ and our studies included very few pregnant women.

Our analysis points to three main conclusions. First, most published studies described populations of people with chronic HBV infection, but did not report data (ie, liver fibrosis staging, HBV DNA level, and ALT) in a consistent format that could be used to examine eligibility for treatment according to different criteria. The adoption of consistent reporting standards in studies of people who are HBsAg positive would facilitate evaluation of the impact of applying different criteria for eligibility. Second, 10% of people with chronic HBV infection across all studies are eligible for treatment based on presence of cirrhosis. Third, using heterogeneous data from studies using different criteria, between a tenth and a quarter of people with HBV infection might be eligible for treatment depending on whether people were recruited in the community or in clinical settings. When applied to the estimated 257 million people with HBV infection worldwide, this would suggest that between 20 million and 64 million people are eligible for treatment. This range could be used as an initial working estimate while awaiting better sources of information.

We propose several actions. First, studies reporting cross-sectional data on people with HBV infection should include estimates of the proportion of participants who are eligible for treatment according to various criteria. Such cross-sectional data would be a useful addition to data from cohorts used to describe natural history and inform guidelines. Descriptions of people with HBV infection should report separately the proportion of cirrhosis (using validated criteria), and the proportion of abnormal ALT and replication (with HBV DNA if possible, or HBeAg if HBV DNA is not available) in patients without cirrhosis. Second, additional efforts are needed to generate disaggregated estimates of the proportion of people eligible for HBV treatment according to the setting where they are identified (ie, population or health-care facilities), age, sex, ethnicity, and geographical areas, as to better understand who is eligible. A better understanding of who is eligible for therapy could also be used to optimise testing approaches, so that population groups with the highest yield of eligible patients could be tested as a priority. Lastly, patients with HBV-related cirrhosis (26 million people, around 10% the 257 million people with HBV infection globally) should be prioritised for treatment initiation to improve survival. Among patients with cirrhosis, according to WHO guidelines, HBV DNA is not needed before treatment initiation, which considerably simplifies management.^4^ This subset of patients would benefit most from treatment.^180^ Implementation of such simplified management algorithms by which diagnosis of HBV infection followed by diagnosis of cirrhosis would lead to immediate treatment would be an urgent, efficient, and effective way to start bridging the major HBV treatment gap.

**This online publication has been corrected. The corrected version first appeared at thelancet.com/gastrohep on January 11, 2021**
