# Correction to *Lancet Gastroenterol Hepatol* 2021; 6: 106–19

**DOI:** 10.1016/S2468-1253(20)30399-X

**Published:** 2021-01-11

*Tan M, Bhadoria A S, Cui F, et al. Estimating the proportion of people with chronic hepatitis B virus infection eligible for hepatitis B antiviral treatment worldwide: a systematic review and meta-analysis.* Lancet Gastroenterol Hepatol *2021; **6:** 106–19—*In this Article, Mingjuan Tan's affiliation should have been listed as Department of Medicine, National University Health System, Singapore. A revised appendix has been uploaded. This correction has been made to the online version as of Jan 11, 2021, and the printed version is correct.

