# Supplementary material for: Estimating the proportion of people with chronic hepatitis B virus infection eligible for hepatitis B antiviral treatment worldwide: a systematic review and meta-analysis
Source: Lancet Gastroenterol Hepatol. 2020 Nov 14;6(2):106–19. doi: 10.1016/S2468-1253(20)30307-1 (PMC7801814; doi:10.1016/S2468-1253(20)30307-1)

THE LANCET
Gastroenterology & Hepatology

Supplementary appendix

This appendix formed part of the original submission and has been peer reviewed. We post it as supplied by the authors.

This online publication has been corrected. The corrected version first appeared at thelancet.com/gastrohep on Jan 11, 2021.

Supplement to: Tan M, Bhadoria A S, Cui F, et al. Estimating the proportion of people with chronic hepatitis B virus infection eligible for hepatitis B antiviral treatment worldwide: a systematic review and meta-analysis. *Lancet Gastroenterol Hepatol* 2021; **6:** 106–19.

Estimating the proportion of persons with chronic HBV infection eligible for hepatitis B antiviral treatment worldwide: A systematic review and meta-analysis

Appendix

Annex 1: Comparison of the treatment eligibility criteria in various HBV treatment guidelines

Various criteria considered in the decision-making process			WHO 2015	APASL 2015	EASL 2012	EASL 2017	AASLD 2016
**Eligibility is based on the combination of abnormal ALT and replication**	**ALT**		Persistently elevated ALT	Persistently elevated ALT (>2 xULN[*])[†]	Persistently elevated ALT (>2 ULN)[*]	ALT > ULN[*‡]	ALT >2 xULN[*]
	**Replication**	**HBV DNA**	> 20,000 UI/ml	> 20,000 UI/ml (HBeAg+) >2,000 UI/ml (HBeAg-)[†]	> 20,000 UI/ml	> 2,000 UI/ml[§]	> 20,000 UI/ml (HBeAg+) >2,000 UI/ml (HBeAg -)
		**HBeAg**	If HBV DNA is not available	Affects the HBV DNA threshold	-	-	Affects the HBV DNA threshold
**Cirrhosis**		**Compensated**	Eligible irrespective of other criteria	Eligible if HBV DNA >2000 IU/ml, irrespective of ALT	Eligible irrespective of other criteria	Eligible irrespective of other criteria	Eligible if HBV DNA >2000 IU/ml, irrespective of ALT
		**Decompensated**		Eligible irrespective of other criteria			
**Family history**			-	Consider biopsy if family history of HCC[**], cirrhosis	-	Eligible if family history of HCC, cirrhosis	Consider treatment if family history of HCC
**Extrahepatic manifestations**			-	-	-	Eligible if extrahepatic manifestations	Consider treatment
**Age > 40**			Age criteria	Consider biopsy if > 35 years	-	-	Consider treatment if > 40 years
**Biopsy**			-	Consider treatment if moderate to severe inflammation or significant liver fibrosis[††]		-	-

[*] ULN: Upper limit of normal
[†] If ALT <2 ULN and DNA> 20,000 UI/ml or if ALT persistently elevated and DNA <20,000 UI/ml, conduct non-invasive assessment of liver fibrosis and monitor every 3 months
[‡] Alternative treatment criteria is if ALT >2 xULN and HBV DNA> 20,000 UI/ml regardless of degree of liver fibrosis
[§] With moderate necroinflammation or liver fibrosis
[**] HCC: Hepatocellular carcinoma
[††] Biopsy should be considered if non-invasive tests suggest evidence of significant liver fibrosis, ALT becomes persistently elevated.

Annex 2: Search strategy

#	Searches	Results
1	"hepatitis b virus"[MeSH] OR "hepatitis b"[MeSH] OR "hepatitis b, chronic"[MeSH] OR "hepatitis b surface antigens"[MeSH] OR "HBV infection"[TW] OR (HBV[TW] AND hepatitis [TW]) OR "hepatitis b"[TW] or "chronic hepatitis b"[tw] or "hbsag"[tw] or "hbs-ag"[tw] or "hbs antigen*"[tw] or "hbs-antigen*"[tw] or "hbv"[tw] or "hepatitis-b"[tw] or "hep b"[tw]	94092
2	"Alanine Transaminase"[Mesh] OR "Alanine Transaminase"[TW] or "alt"[TW] or "alat"[TW] or "gpt"[TW] OR "Liver Cirrhosis"[Mesh] OR "liver cirrhosis"[TW] OR "liver cirrhoses"[TW] OR "hepatic cirrhoses"[TW] OR "hepatic cirrhosis"[TW] OR "liver fibroscan"[TW] OR "liver biopsy"[TW] OR "liver ultrasound"[TW] OR "liver fibroses"[TW] OR "liver fibrosis"[TW] OR "hepatic fibrosis"[TW] OR ("Liver"[Mesh] AND "fibrosis"[mesh]) OR "HBV DNA"[TW] OR "HBV-DNA"[TW] OR "Viral Load"[Mesh] OR "viral load"[TW]	201914
3	"epidemiologic methods"[mesh] OR "Comparative Study "[Publication Type] OR "OUTCOME AND PROCESS ASSESSMENT (HEALTH CARE)"[Mesh] OR "statistics and numerical data "[Subheading] OR "Evaluation Studies "[Publication Type] OR "meta analysis "[Publication Type] or "multicenter study "[Publication Type] OR incidence [TIAB] OR surveillance [TIAB] OR prevalence [TIAB] OR "epidemiology" [subheading] OR "Health Care Evaluation Mechanisms"[Mesh] OR morbidity [TIAB] OR systematic [SB] OR burden [TW] OR screening [TIAB] OR characteristics [TIAB]	8973941
4	#1 AND #2 AND #3	15064
Filter	Publication date from 2007/01/01	8534

Annex 3: Title and abstract screening keywords

Title screening

Include	Exclude
• Population of adult human subjects who are HbsAg positive or who are referred to as having "chronic hepatitis B", including clinical trials and serological studies	• Study participants do not have hepatitis B<br>• Study participants belong to subset/s of persons with hepatitis B<br>• Study participants are restricted to those with a primary condition other than hepatitis B (e.g. malignancy, autoimmune condition, hemodialysis)<br>• Basic science studies (In vitro only)<br>• Study type: case report, meta-analysis or review, studies on diagnostic methods (e.g. evaluation of blood donation screening methods), opinion or editorial, knowledge or qualitative survey, cost analysis

Abstract screening

Include	Exclusion
• See above keywords and criteria<br>• Studies that include information on one of several of the following:<br>a) Fibrosis and/or cirrhosis status<br>b) Data on abnormal ALT levels<br>c) Data on HBV DNA level<br>d) Data on the number of subjects who are HBeAg positive	• See above keywords and criteria<br>• RCTs in which all subjects are already known to be eligible for hepatitis B treatment<br>• Studies (e.g. serological studies) which do not provide information on any of the characteristics (besides HbsAg) that allows assessment of treatment eligibility

Annex 4: Data extraction form

Hepatitis B Systematic Review - Data Extraction Form

Part 1: Study details

Study number

Study subset

Final status: Included?

Study title

If no, reason for exclusion

Data extraction completed

Other reason for exclusion

Year published

First author

Author contacted

Journal

Author email

DOI

Date contacted

PubMed ID

Language of publication

- English
- French
- Chinese
- Japanese
- Korean
- Portugese
- Russian

Part 2: Study population characteristics

Start date of study

End date of study

Type of study
- Cohort
- Case control
- Cross sectional

Logistics of data collection
- Prospective
- Retrospective
- Cross sectional

Country

City (if applicable)

WHO region of study

Immigration status of participants
- Non immigrant
- Immigrant
- Refugee
- Asylum Seeker
- Native and immigrant
- No information or not

WHO region of participants
- AFRO
- SEARO
- EMRO
- WPRO
- EURO
- Unknown
- PAHO

Prevalence of Coinfections Reported

HDV

HDV percentage

HCV

HCV percentage

HIV

HIV percentage

Other characteristics reported

Injection drug use

IV percentage

Alcohol use

Alcohol percentage

TM percentage

Hepatotoxic traditional medicines

Prev treatment percentage

Previous hepatitis B treatment

Mean age

Median age

Age range low

Age range high

Percentage Male

Part 3: Risk of bias / Study quality assessment

I. Selection bias

Recruitment of study participants

- ☐ Clinic
- ☐ Blood bank
- ☐ Hospital
- ☐ Community statistical sampling
- ☐ Community nonstatistical sampling
- ☐ Special population
- ☐ Other

Specify [ ]

No. of HbsAg positive subjects

[ ]

Non response proportion

[ ]

Dropout proportion

[ ]

II. Information bias

Coinfections

- ☐ HIV
- ☐ HCV
- ☐ HDV

Analysis or matching done for major confounders

[▾]

Main design flaws

[ ]

Cirrhosis/fibrosis

Subjects assessed for cirrhosis or fibrosis

[▾]

If yes, mode of diagnosis of cirrhosis/fibrosis:

- ☐ Clinical
- ☐ Ultrasound
- ☐ Fibroscan
- ☐ Biopsy

Diagnosis of cirrhosis or fibrosis done in same way for all participants

- ○ Yes
- ○ No
- ○ Not specified

Part 4: Study data

Variables for which info provided in required format

- ☐ HbeAg
- ☐ HBV DNA
- ☐ ALT
- ☐ Cirrhosis and/or fibrosis

Variables on which info in other format

- ☐ HbeAg
- ☐ HBV DNA
- ☐ ALT
- ☐ Cirrhosis and/or fibrosis

Data cross tabulated

Hepatitis B Markers

Number of participants screened for HbsAg

Number of participants positive for HBsAg

Proportion of participants positive for HBsAg

HBV DNA

Number of participants screened for HBV DNA

HBV DNA > 2000 IU/ml

Proportion >2000

HBV DNA >20,000 IU/ml

Proportion > 20,000

HBV DNA above other threshold

Specify other threshold

Cirrhosis/fibrosis data

Number of participants screened for fibrosis or cirrhosis

F2 (significant fibrosis)

F3 (severe fibrosis)

% sig-severe fibrosis

F4 (cirrhosis)

Proportion with cirrhosis

HbeAg

Number of participants screened for HbeAg

Number positive for HbeAg

% positive HbeAg

ALT

Number of participants screened for ALT

ULN as defined by study

ALT > ULN

% ALT > ULN

ALT > 2x ULN

% ALT > 2x ULN

Participants have ALT followup

Hepatitis B genotype

Reported

Study reports treatment eligibility number or percentage

If yes, guideline/s used:

☐ WHO 2015

If yes, reported number

If yes, reported percentage

☐ EASL 2017

If yes, reported number

If yes, reported percentage

☐ EASL 2012

If yes, reported number

If yes, reported percentage

☐ AASLD 2015

If yes, reported number

If yes, reported percentage

☐ APASL 2015

If yes, reported number

If yes, reported percentage

Highest reported percentage

Lowest reported percentage

Percentage which actually received treatment

☐ Country

If yes, reported number

If yes, reported percentage

☐ Other guideline

Specify:

Reported number

Reported percentage

Annex 5: PRISMA checklist

Section/topic	#	Checklist item	Place reported
**TITLE**			
Title	1	Identify the report as a systematic review, meta-analysis, or both.	Title
**ABSTRACT**			
Structured summary	2	Provide a structured summary including, as applicable: background; objectives; data sources; study eligibility criteria, participants, and interventions; study appraisal and synthesis methods; results; limitations; conclusions and implications of key findings; systematic review registration number.	First page
**INTRODUCTION**			
Rationale	3	Describe the rationale for the review in the context of what is already known.	Introduction
Objectives	4	Provide an explicit statement of questions being addressed with reference to participants, interventions, comparisons, outcomes, and study design (PICOS).	Introduction
**METHODS**			
Protocol and registration	5	Indicate if a review protocol exists, if and where it can be accessed (e.g., Web address), and, if available, provide registration information including registration number.	End of methods section
Eligibility criteria	6	Specify study characteristics (e.g., PICOS, length of follow-up) and report characteristics (e.g., years considered, language, publication status) used as criteria for eligibility, giving rationale.	Methods section
Information sources	7	Describe all information sources (e.g., databases with dates of coverage, contact with study authors to identify additional studies) in the search and date last searched.	Methods section
Search	8	Present full electronic search strategy for at least one database, including any limits used, such that it could be repeated.	Annex 2
Study selection	9	State the process for selecting studies (i.e., screening, eligibility, included in systematic review, and, if applicable, included in the meta-analysis).	P4-5, figure 2
Data collection process	10	Describe method of data extraction from reports (e.g., piloted forms, independently, in duplicate) and any processes for obtaining and confirming data from investigators.	Methods section
Data items	11	List and define all variables for which data were sought (e.g., PICOS, funding sources) and any assumptions and simplifications made.	Annex 4
Risk of bias in individual studies	12	Describe methods used for assessing risk of bias of individual studies (including specification of whether this was done at the study or outcome level), and how this information is to be used in any data synthesis.	Methods section
Summary measures	13	State the principal summary measures (e.g., risk ratio, difference in means).	Methods section
Synthesis of results	14	Describe the methods of handling data and combining results of studies, if done, including measures of consistency (e.g., I^2) for each meta-analysis.	Methods section

Section/topic	#	Checklist item	Reported on page #
Risk of bias across studies	15	Specify any assessment of risk of bias that may affect the cumulative evidence (e.g., publication bias, selective reporting within studies).	Figure 3-7 (Appendix)
Additional analyses	16	Describe methods of additional analyses (e.g., sensitivity or subgroup analyses, meta-regression), if done, indicating which were pre-specified.	Methods section
**RESULTS**			
Study selection	17	Give numbers of studies screened, assessed for eligibility, and included in the review, with reasons for exclusions at each stage, ideally with a flow diagram.	Figure 1
Study characteristics	18	For each study, present characteristics for which data were extracted (e.g., study size, PICOS, follow-up period) and provide the records.	Database
Risk of bias within studies	19	Present data on risk of bias of each study and, if available, any outcome level assessment (see item 12).	Table 1-3
Results of individual studies	20	For all outcomes considered (benefits or harms), present, for each study: (a) simple summary data for each intervention group (b) effect estimates and confidence intervals, ideally with a forest plot.	Table 1-4 Figure 2, 3, 8
Synthesis of results	21	Present results of each meta-analysis done, including confidence intervals and measures of consistency.	Results section
Risk of bias across studies	22	Present results of any assessment of risk of bias across studies (see Item 15).	Figure 3-7 (Appendix)
Additional analysis	23	Give results of additional analyses, if done (e.g., sensitivity or subgroup analyses, meta-regression [see Item 16]).	Figure 2-8
**DISCUSSION**			
Summary of evidence	24	Summarize the main findings including the strength of evidence for each main outcome; consider their relevance to key groups (e.g., healthcare providers, users, and policy makers).	Beginning of discussion
Limitations	25	Discuss limitations at study and outcome level (e.g., risk of bias), and at review-level (e.g., incomplete retrieval of identified research, reporting bias).	Discussion: Dedicated paragraph
Conclusions	26	Provide a general interpretation of the results in the context of other evidence, and implications for future research.	Last two paragraph of the discussion
**FUNDING**			
Funding	27	Describe sources of funding for the systematic review and other support (e.g., supply of data); role of funders for the systematic review.	Summary

Figure 3: Forest plot documenting pooled estimates of the proportion of persons with cirrhosis (n-104) *

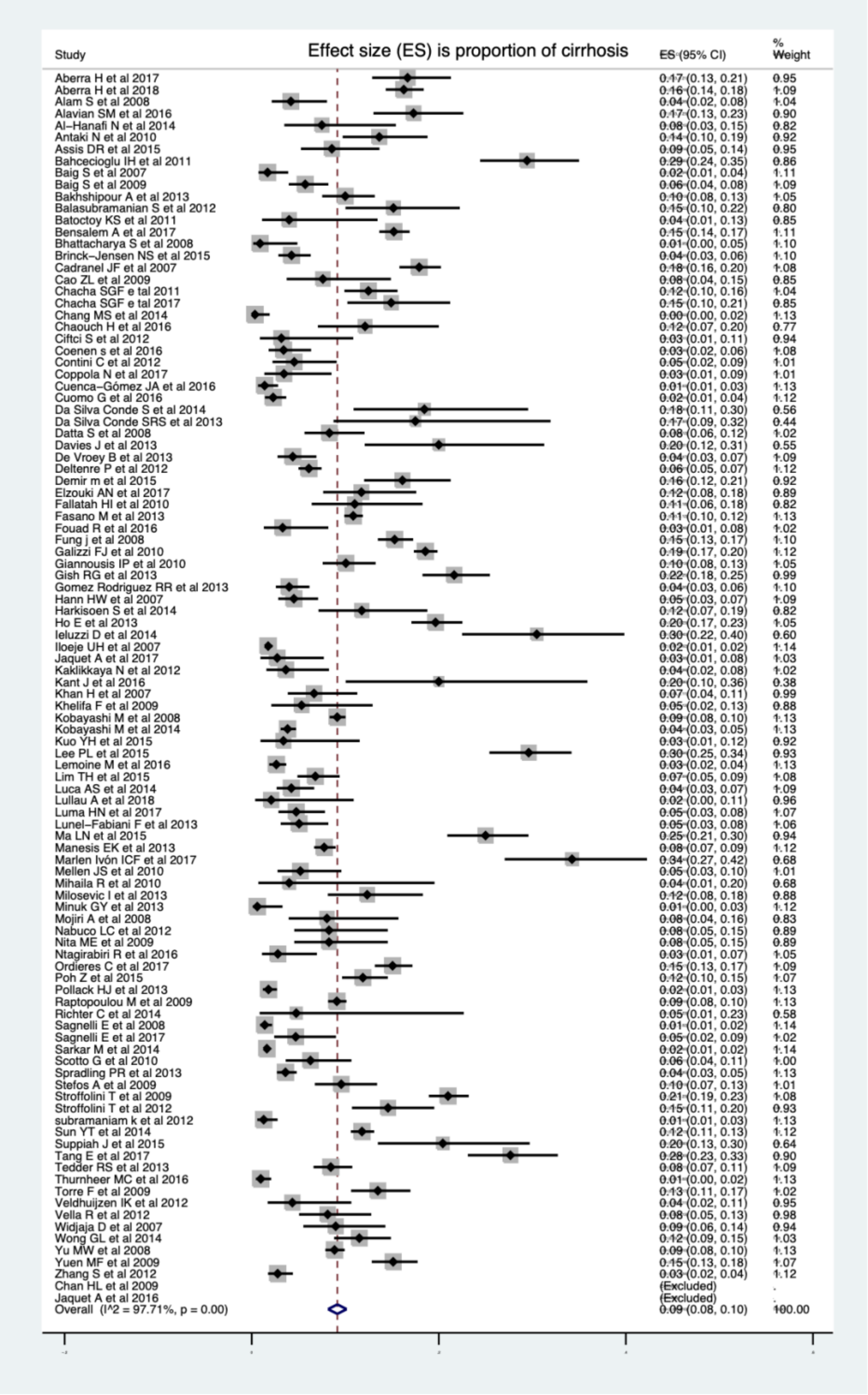

* Pooled Effect Size = 9% (8-10%); HeterogeneityϗΧ2 = 4404.95 (d.f. = 101) p<0.001 I2 (variation in ES attributable to heterogeneity) = 97.71%

Figure 4: Bubble plot displaying the relationship between assessment method and the proportion of cirrhosis in the studies identified (n=104)*

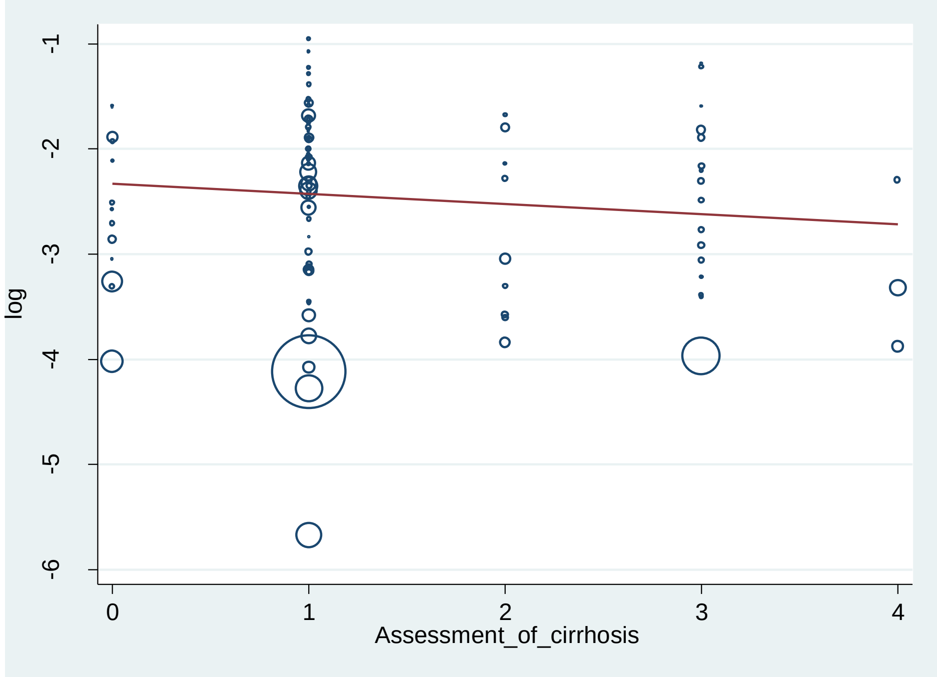

Key: 0: Data not available; 1: Biopsy; 2: Fibroscan; 3: ultrasound; 4: Clinical criteria (p=0.253)

*Bubble plot is a graphical representation of the results of the meta-regression. The red line represents the regression line. Individuals bubble represents studies. The size of the bubble represents the weight of the study that comes from the sample size. The Y axis is log of effect size. The x axis displays various methods of assessment of liver fibrosis as per the key. The P value (p=0.253) represents the statistical significance of the relationship between the variate and the effect size.

Figure 5: Funnel plot with pseudo 95% confidence limits for the proportion of subjects with cirrhosis (n=104)*

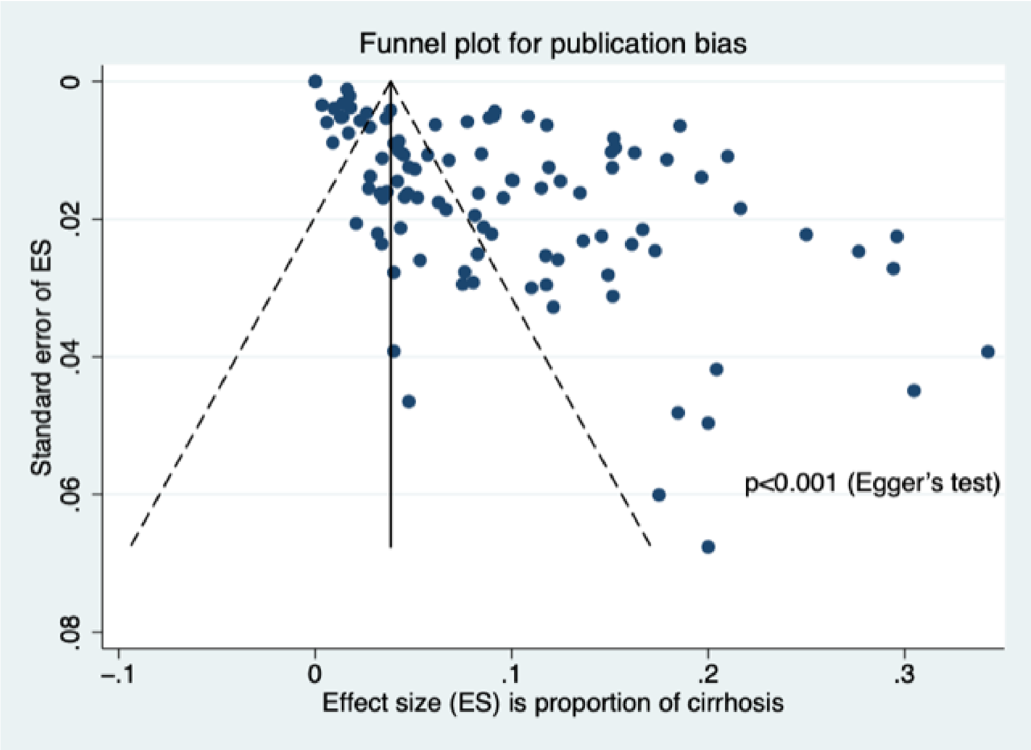

---

* The funnel plot depicts publication bias in the studies that were included. The dots positioned in the triangle represents studies with no publication bias. Studies outside the triangle may have a publication bias. Those on the right of the triangle represent published studies that report a higher proportion of cirrhosis. Those on the left of the triangle represent studies that report a lower proportion of cirrhosis.

Figure 6: Bubble plot with regression line for possible confounders for the proportion of persons eligible for treatment (A, B , C are for studies done in health care facilities and D, E, F are for studies done in community setting)*

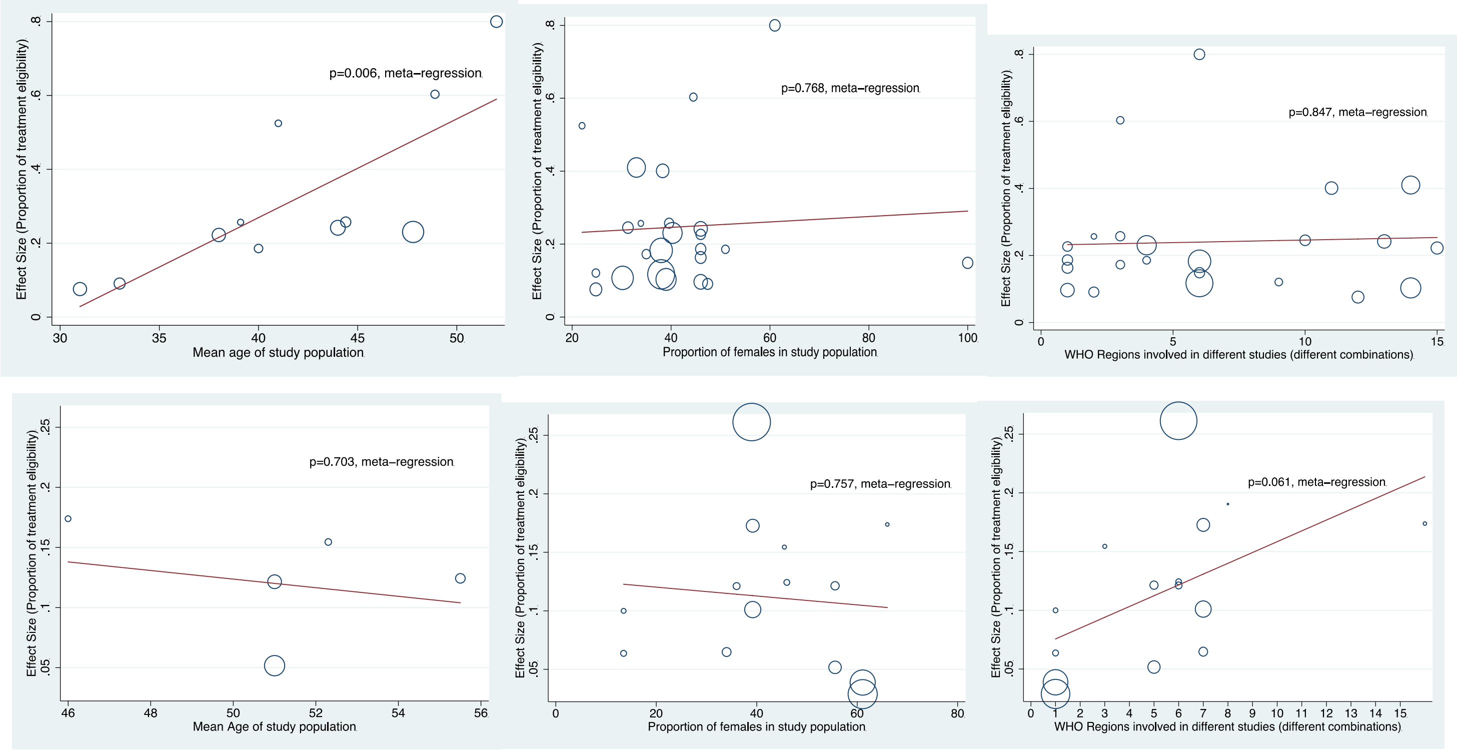

* The p values test the statistical significance of the association between the variable of interest and the proportion of person eligible for treatment. For example in bubble plot 'A', p=0.006 for mean age. This indicates a statistically significant association between the mean age of study population and the proportion of persons who were eligible.

Figure 7: Funnel plots for estimates of treatment eligibility according to any guidelines. First plot is for studies conducted in health care facilities and second one for studies conducted in community

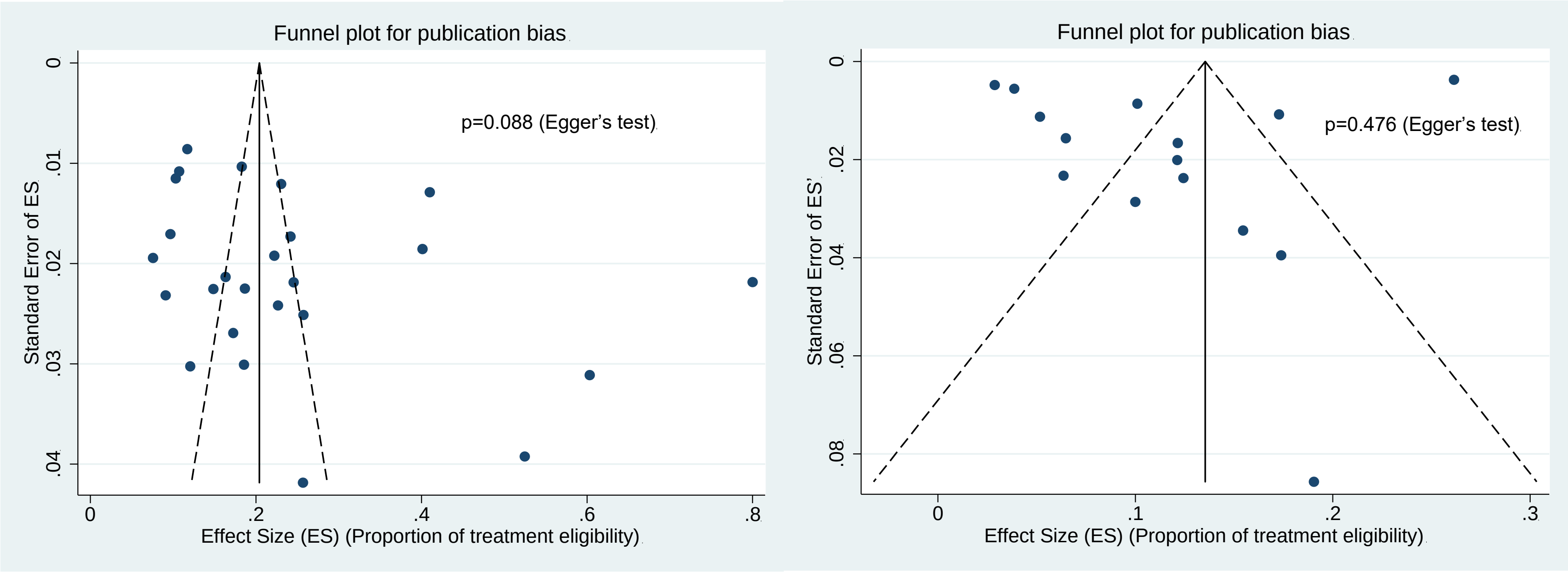

Figure 8: Random effect model showing forest plot with pooled estimates of cirrhosis. First plot is for studies conducted in health care facilities (n=80) and second one for studies conducted in community (n=24)

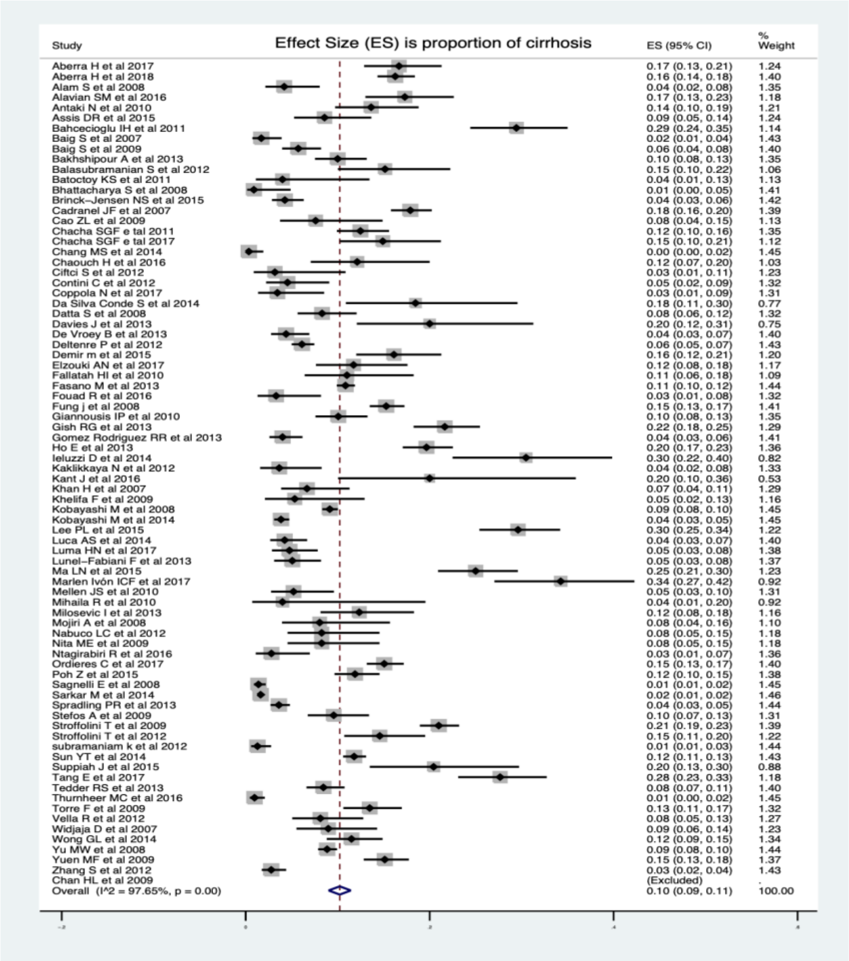

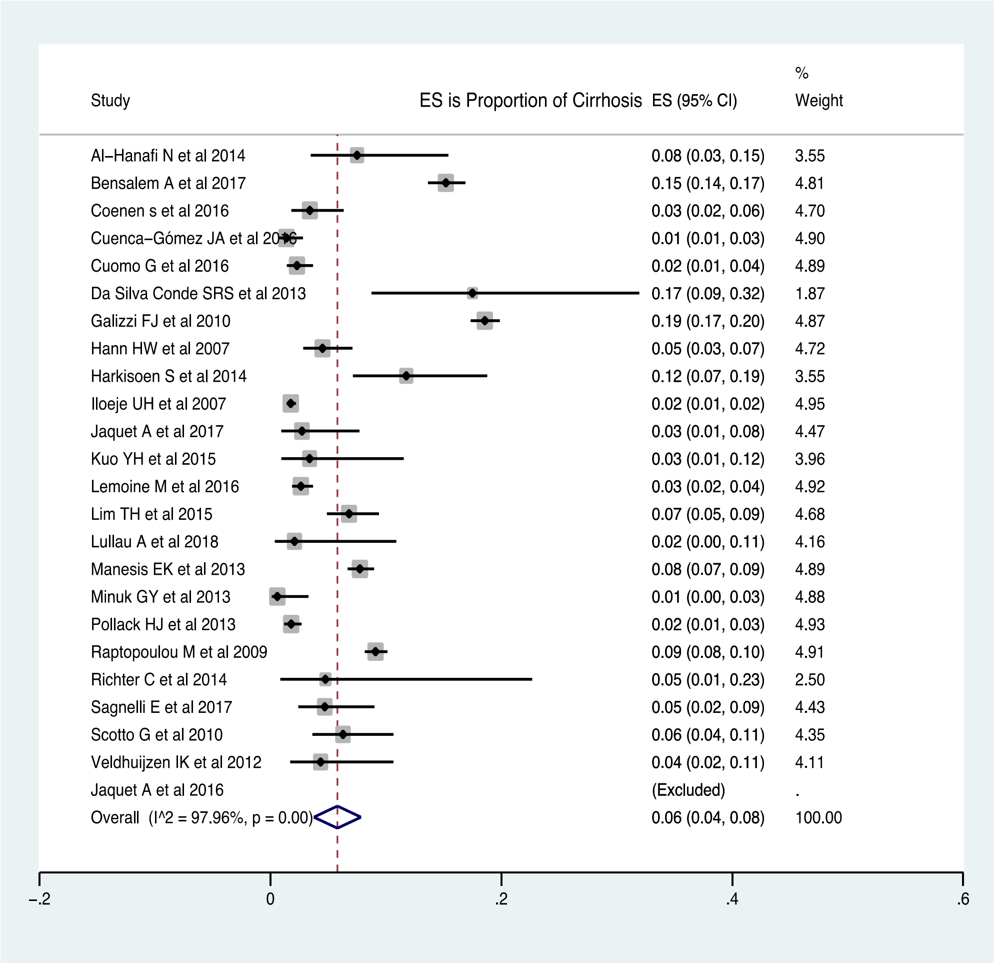

Supplement: Supplementary appendix [file mmc1.pdf]
